# P-48. How Many Lumens Should Be Cultured? Evaluating the Yield of Culturing All Lumens in Febrile Neutropenic Children with Cancer

**DOI:** 10.1093/ofid/ofaf695.277

**Published:** 2026-01-11

**Authors:** M U A Y A D ALALI

**Affiliations:** Indiana university, Carmel, Indiana

## Abstract

**Background:**

Culturing all central venous catheter (CVC) lumens is standard in pediatric febrile neutropenia (FN) to optimize catheter-related bloodstream infection (CRBSI) diagnosis. However, this approach increases blood volume use and bottle requirements, raising concerns during shortages like in 2024. Data on the risk of missed CRBSI diagnoses when not all lumens are cultured are limited

**Table:**
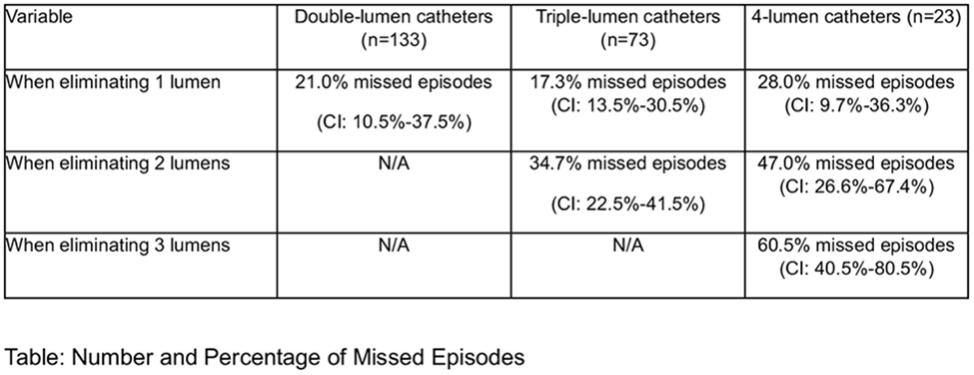
Number and Percentage of Missed Episodes

**Methods:**

This single-center, retrospective study at Riley Hospital for Children (2016–2023) evaluated febrile neutropenia (FN) episodes to determine the proportion of catheter-related bloodstream infections (CRBSI) missed when not culturing all lumens of multi-lumen catheters. Only microbiologically proven CRBSI episodes with simultaneous cultures from all lumens were included. Analysis focused on double-, triple-, and quadruple-lumen catheters, estimating missed diagnosis rates using statistical modeling and 1,000 simulated samples, with significance set at p< 0.05

**Results:**

A total of 377 proven CRBSI episodes were identified from 2,648 FN episodes in 223 patients. After excluding cases where blood cultures were drawn from single-lumen catheters, 229 CRBSI episodes from 163 patients with multi-lumen catheters were included. This included 133 double-lumen, 73 triple-lumen, and 23 quadruple-lumen catheter episodes.

Eliminating cultures from one lumen resulted in missed diagnoses for 21%, 17.3%, and 28% of CRBSI episodes in double-lumen, triple-lumen, and quadruple-lumen catheters, respectively. If two lumens were excluded, the missed diagnosis rate increased to 34.7% for triple-lumen and 47% for quadruple-lumen catheters (see table)

**Conclusion:**

Eliminating cultures from even one lumen significantly increases the risk of missed CRBSI diagnoses and should be carefully considered, especially during resource-limited periods, to maintain diagnostic accuracy and patient care

**Disclosures:**

All Authors: No reported disclosures

